# Energy Efficiency of D2D Multi-User Cooperation

**DOI:** 10.3390/s17040697

**Published:** 2017-03-28

**Authors:** Zufan Zhang, Lu Wang, Jie Zhang

**Affiliations:** 1 School of Communication and Information Engineering, Chongqing University of Posts and Telecommunications, Chongqing 400065, China; zhangzf@cqupt.edu.cn (Z.Z.); wanglu19931206@gmail.com (L.W.); 2 Chongqing Key Laboratory of Mobile Communication, Chongqing 400065, China

**Keywords:** D2D communication, multiple user cooperation, energy efficiency, outage probability

## Abstract

The Device-to-Device (D2D) communication system is an important part of heterogeneous networks. It has great potential to improve spectrum efficiency, throughput and energy efficiency cooperation of multiple D2D users with the advantage of direct communication. When cooperating, D2D users expend extraordinary energy to relay data to other D2D users. Hence, the remaining energy of D2D users determines the life of the system. This paper proposes a cooperation scheme for multiple D2D users who reuse the orthogonal spectrum and are interested in the same data by aiming to solve the energy problem of D2D users. Considering both energy availability and the Signal to Noise Ratio (SNR) of each D2D user, the Kuhn-Munkres algorithm is introduced in the cooperation scheme to solve relay selection problems. Thus, the cooperation issue is transformed into a maximum weighted matching (MWM) problem. In order to enhance energy efficiency without the deterioration of Quality of Service (QoS), the link outage probability is derived according to the Shannon Equation by considering the data rate and delay. The simulation studies the relationships among the number of cooperative users, the length of shared data, the number of data packets and energy efficiency.

## 1. Introduction

As multimedia communication develops, traditional cellular networks will not be able to handle large amounts of data and support high-speed transmission. D2D communication can help enhance data rate, system capacity and spectrum efficiency through short-range communication among users directly by reusing the spectrum of cellular network [1,2,3], which gives users a better communication experience than the traditional cellular network.

Furthermore, D2D pairs can build a self-organizing system by establishing connections with the help of a cellular network. Due to flexibility, multiple D2D users who want to receive the same data from an Evolved Node B (eNB) can organize a cluster, and a cluster head is chosen to receive the data from the eNB to share with cluster members by dividing the data into multiple data packets, which is the mesh D2D service introduced in Reference [4]. Regarded as a proximity service, D2D services are sufficiently flexible to adapt to various complex heterogeneous networks in the future.

D2D communication is an important part of heterogeneous networks. The spectrum efficiency, throughput and energy efficiency can be improved by interference suppression, resource allocation and relay selection schemes [5,6,7,8]. Many studies show that most researches on short-range communication systems focus on improving communication reliability and data rate among users [9,10,11]. As D2D users communicate directly with each other through short-range communication (which consumes a small amount of energy due to the short distance [12]), whether the users can provide enough energy for the packet transmission or not is usually ignored. In fact, though the cooperation among a large number of D2D users can effectively enhance the spectrum efficiency and throughput of heterogeneous networks [13], the amount of consumed energy of each D2D user obviously rises with the number of cooperative users. Hence, it is necessary to take the energy availability of each user into account when there are multiple cooperative users.

Traditional ways of choosing relaying users were based on the maximum SNR [14,15], which resulted in high energy consumption. On one hand, particular users are chosen by multiple users simultaneously, which results in energy waste. On the other hand, particular users will be chosen at different times, which will cost these particular users a large amount of energy. Hence, it is not appropriate to choose relaying D2D users based on the maximum SNR when there are multiple cooperative users.

In the D2D cluster, some D2D users will consume additional energy as the relaying of other D2D users to receive data from the base station and then relay the data to corresponding D2D user. Which will cost these part of relaying D2D users a large amount of energy. In order to avoid excessive energy consumption of some D2D users and decrease of the system lifetime, this paper reduces the energy consumption of the single D2D user by preventing a D2D user from being chosen as a relaying by multiple D2D user at the same time and always being chosen at different times. So, we restrict that the relaying D2D user only relays data for one of the other D2D users. Then the key is how to match multiple relaying D2D users with the rest of multiple D2D users. The Kuhn-Munkres algorithm is a classic algorithm in graph theory to find the optimal match of weighted bipartite graph. Motivated by the application of the Kuhn-Munkres algorithm in Reference [16], where the Kuhn-Munkres algorithm was introduced to solve the matching problem of maximizing transmission capacity, this paper adopts the Kuhn-Munkres algorithm to deal with this issue. Hence, the D2D multi-user cooperation issue is transformed into a MWM problem.

This paper aims to solve the energy problem of D2D users when there are multiple D2D users cooperating. In the D2D communication system, D2D users who want to receive the same data form a cluster by a self-organizing connection with the help of a cellular network. D2D users use different spectrums to provide mesh D2D service to avoid co-channel interference. The main contributions of this paper are as follows:

1. A D2D multi-user cooperation scheme is proposed, choosing the relaying D2D users by considering both energy availability and the SNR of each D2D user. Each relaying D2D user can be prevented from being chosen by multiple D2D users at both the same time and at different times with the help of the Kuhn-Munkres algorithm.
2. To guarantee high data rate and low delay, the outage probability of the D2D link is derived according to the Shannon Equation and the system energy efficiency is analyzed combined with the outage probability of a D2D link.
3. To achieve D2D multi-user cooperation with high energy efficiency in different communication environments, the relationships among the number of cooperative users, the length of shared data, the number of data packets and the energy efficiency are studied in the simulation.

## 2. Cooperation of D2D Multiple Users

**Figure 1:**
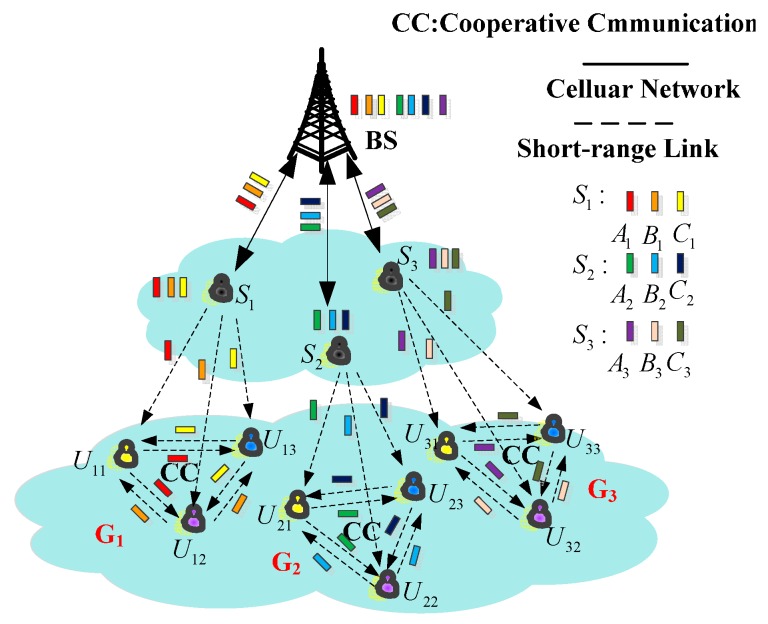
D2D multi-user cooperation within a heterogeneous network.

In a heterogeneous network which consists of a cellular and D2D network, multiple D2D users who are interested in the same data form a cluster with the help of a cellular network. The cooperation system is shown in Figure 1, where *G*_1_, *G*_2_, *G*_3_ are different clusters and *S*_1_, *S*_2_, *S*_3_ are their cluster heads. After receiving the data, the cluster head divides the data into several data packets and sends parts of them to cluster members, than the cluster members cooperate with each other to share what they have received from the cluster head.

Due to the limitation of energy, we have to ensure that each D2D user is prevented from being chosen as the relaying user by multiple D2D users at both the same time and at different times. Meanwhile, the length and the number of data packets can be changed according to various communication environments to reach a high energy efficiency.

**Figure 2:**
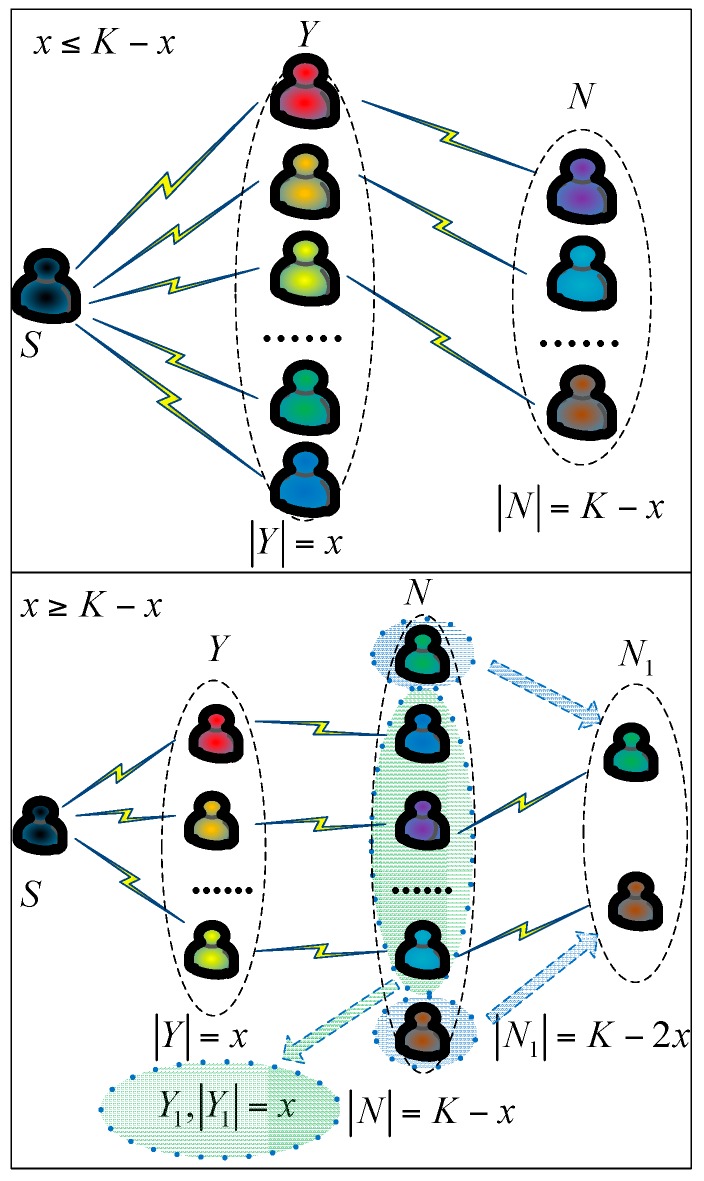
Cooperation mode of multiple D2D users within a cluster.

D2D multi-user cooperation within a cluster is shown in Figure 2. |*Y*| is the number of set *Y*. We suppose that there are *K* + 1 D2D users within a cluster which included one cluster head *S* and *K* cluster members. *S* chooses *x* relaying D2D users from *K* cluster members to receive data packet and *x* changes according to the various communication environments. We suppose that *K* = *ax* + *b*, where *a, b* are integers and a≥1,0≤b≤x. Hence there are four types of cooperation situations.

1. a=1,b=0, namely, *K = x*. *S* has enough energy to broadcast data packet to *K* D2D users and the SNR of each D2D user is good enough to decode the data packet.
2. a=1,0<b<x, namely, *K = x + b*. The chosen *x* relaying D2D users form set $Y_{1}\left(y_{1}^{1},y_{2}^{1},\cdot \cdot \cdot y_{x}^{1}\right)$ and the rest of *K – x = b* D2D users form set $N_{1}\left(n_{1}^{1},n_{2}^{1},\cdot \cdot \cdot ,n_{b}^{1}\right)$. If we regard D2D users as nodes and communications between D2D users as lines, set $Y_{1}\left(y_{1}^{1},y_{2}^{1},\cdot \cdot \cdot y_{x}^{1}\right)$ and $N_{1}\left(n_{1}^{1},n_{2}^{1},\cdot \cdot \cdot ,n_{b}^{1}\right)$ can form a bipartite graph $G_{1}\left(Y_{1},N_{1}\right)$. First, *S* broadcasts the data packet to *Y*_1_ before the D2D users of *N*_1_ pair with the relaying D2D users of *Y*_1_ with a one to one match.
3. a>1,b=0, namely, *K = ax*. *S* first broadcasts the data packet to chosen *x* relaying D2D users. These chosen relaying D2D users form set $Y_{1}\left(y_{1}^{1},y_{2}^{1},\cdot \cdot \cdot y_{x}^{1}\right)$ and the rest of (*a* − 1)*x* D2D users form set $N_{1}\left(n_{1}^{1},n_{2}^{1},\cdot \cdot \cdot ,n_{(a-1)x}^{1}\right)$. Next, the relaying D2D users are chosen from the set whose members have not yet received data packet. For example, *x* D2D users of *N*_1_ will be chosen by *Y*_1_ as relaying D2D users and relay to the rest (up to *x* D2D users).
4. a>1,0<b<x, namely, *K = ax + b*. This cooperation situation combines Situation 3 and Situation 2.

The cooperation process has two parts: (1). *S* chooses *x* relaying D2D users; and (2). *x* relaying D2D users pair with the rest of the D2D users and relay the data packet to them.

In the first part, in order to ensure that the chosen *x* relaying D2D users have enough energy to relay the data packet, *S* chooses *v* D2D users whose energy is higher than energy threshold *I* and these *v* D2D users form set *V*:

(1) $$V=\left\{U_{j}|B_{j}\geq I,1\leq j\leq v,x\leq v\leq K\right\}$$

where $B_{j}$ is the energy of D2D user $U_{j}$. We supposed that the channel state information (CSI) is known by each D2D user. *S* chooses *x* relaying D2D users from *V* according to remaining energy and the channel qualities of the candidates. We let SNR represent the channel quality, where $SNR_{1},\cdot \cdot \cdot ,SNR_{v}$ are SNRs of members of *V*, and the bit error rates can be calculated with SNRs according to the modulation method. Supposing that $P_{b}^{1},\cdot \cdot \cdot ,P_{b}^{v}$ are respective bit error rates and $E_{d}^{1},\cdot \cdot \cdot ,E_{d}^{v}$ are consumed energy by *S* for sending the data packe D(bit) to *V*, we rank $E_{d}^{1},\cdot \cdot \cdot ,E_{d}^{v}$ from the smallest to the largest and obtain the ranked sequence $E_{1},E_{2},\cdot \cdot \cdot ,E_{v}$. The former *x* D2D users are chosen as relaying if $E_{x-sum}\leq B_{s}$, where $E_{x-sum}=E_{1}+\cdot \cdot \cdot +E_{x},1\leq x\leq v$.

In the second part, the chosen *x* relaying D2D users of *Y*_1_ choose *b* (*K = x + b*) or *x* (*K = ax + b, a >* 1) D2D users from *N*_1_ with a one to one match. The future chosen *b* D2D users do not relay the same data packet, while the future chosen *x* D2D users do. Hence, we will analyze the outcome in two situations:

**A.** a=1,0≤b<x, $Y_{1}\left(y_{1}^{1},y_{2}^{1},\cdot \cdot \cdot y_{x}^{1}\right)$ will pair with $N_{1}\left(n_{1}^{1},n_{2}^{1},\cdot \cdot \cdot ,n_{b}^{1}\right)$. It is possible for each member of *Y*_1_ to be chosen by multiple D2D users of *N*_1_ and each member of *N*_1_ will receive the same data packet from multiple relaying D2D users of *Y*_1_, which will cost large amounts of energy for D2D users. In order to save the energy of each D2D user without the deterioration of QoS, the Kuhn-Munkres algorithm is adopted to pair *Y*_1_ and *N*_1_ with a one to one match. 

The D2D users and their cooperation can be regarded as dots and lines in the bipartite graph. *Y*_1_ and *N*_1_ form a bipartite graph $G_{1}\left(Y_{1},N_{1}\right)$. After paired by Kuhn-Munkres algorithm, the adjacency matrix of the paired D2D users can be expressed as:

(2) $$A_{1}={\left[\begin{array}{ccc} a_{1,1} & \cdot \cdot \cdot & a_{1,K-x}\\ \cdot \cdot \cdot & \cdot \cdot \cdot & \cdot \cdot \cdot \\ a_{x,1} & \cdot \cdot \cdot & a_{x,K-x} \end{array}\right]}_{\left(x\cdot \left(K-x\right)\right)}$$

where $a_{i,j}\in \left\{0,1\right\},1\leq i\leq x,1\leq j\leq K-x$, and $a_{i,j}=1$ means that $y_{i}^{1}$ is the relaying D2D user of $n_{j}^{1}$. The SNRs of cooperation links among *Y*_1_ and *N*_1_ can be calculated according to the CSI, which can be expressed as a matrix:

(3) $$R={\left[\begin{array}{ccc} \gamma_{1,1} & \cdot \cdot \cdot & \gamma_{1,K-x}\\ \cdot \cdot \cdot & \cdot \cdot & \cdot \cdot \cdot \\ \gamma_{x,1} & \cdot \cdot \cdot & \gamma_{x,K-x} \end{array}\right]}_{\left(x\cdot \left(K-x\right)\right)}$$

where $\gamma_{i,j},1\leq i\leq x,1\leq j\leq K-x$ represents the SNR of D2D user $U_{j}$ whose relaying D2D user is $U_{i}$. Next, the SNRs of each row is ranked from the largest to the smallest and given labels K−x,⋅⋅⋅,1, which means that the larger the SNR the bigger the label is given to the respective D2D user. Thus, the corresponding label matrix can be expressed as:

(4) $$L={\left[\begin{array}{ccc} l_{1,1} & \cdot \cdot \cdot & l_{1,K-x}\\ \cdot \cdot \cdot & \cdot \cdot \cdot & \cdot \cdot \cdot \\ l_{x,1} & \cdot \cdot \cdot & l_{x,K-x} \end{array}\right]}_{\left(x\cdot \left(K-x\right)\right)}$$

where $l_{i,j}$ represents the weight of line which connects $U_{i}$ and $U_{j}$ in bipartite graph $G_{1}\left(Y_{1},N_{1}\right)$. The larger weight means better communication quality. Hence, we adopt the Kuhn-Munkres algorithm to match *Y*_1_ and *N*_1_ with the maximum SNR. As the Kuhn-Munkres algorithm requires that *Y*_1_ and *N*_1_ should be equal, *x* − (*K* − *x*) zeros are added to each row of label matrix *L* to make a square matrix $L^{\prime }$:

(5) $$L^{\prime }={\left[\begin{array}{cc} \begin{array}{ccc} l_{1,1} & \cdot \cdot \cdot & l_{1,K-x}\\ \cdot \cdot \cdot & \cdot \cdot \cdot & \cdot \cdot \cdot \\ l_{x,1} & \cdot \cdot \cdot & l_{x,K-x} \end{array} & \underset{\begin{array}{l} \mathrm{each}\;\mathrm{column}\;\mathrm{is}\;0\;\mathrm{from}\\ \mathrm{column}\;K-x+1\;\mathrm{to}\;\mathrm{column}\;x \end{array}}{\underset{︸}{\begin{array}{ccc} 0 & \cdot \cdot \cdot & 0\\ \cdot \cdot \cdot & \cdot \cdot \cdot & \cdot \cdot \cdot \\ 0 & \cdot \cdot \cdot & 0 \end{array}}} \end{array}\right]}_{\left(x\cdot x\right)}$$

where $l_{i,j}$, 1≤i≤x, 1≤j≤x, $l_{i,j}=0$ K−x<j≤x.

**B.** a>1,0≤b<x, the size of $Y_{1}\left(y_{1}^{1},y_{2}^{1},\cdot \cdot \cdot y_{x}^{1}\right)$ is smaller than that of $N_{1}\left(n_{1}^{1},n_{2}^{1},\cdot \cdot \cdot ,n_{(a-1)x+b}^{1}\right)$. The *x* D2D users chosen by *Y*_1_ from *N*_1_ will be the relaying D2D users of the rest of (*a* − 2)*x* + *b* D2D users of *N*_1_. *Y*_1_ and *N*_1_ are supposed to form bipartite graph. $G_{2}\left(Y_{1},N_{1}\right)$. Hence, before matching *Y*_1_ and *N*_1_ based on SNRs, it is necessary to consider the energy of the relaying candidates. $v^{\prime }$ D2D users whose maintaining energy $B_{i}$ is bigger than energy threshold $I^{\prime }$are chosen from *N*_1_ to form set $V^{\prime }$:

(6) $$V^{\prime }=\left\{U_{i}|B_{i}\geq I^{\prime },1\leq i\leq v^{\prime },x\leq v^{\prime }\leq K-x\right\}$$

Then, *Y*_1_ is matched with $V^{\prime }$ according to the SNRs as in Situation A. The corresponding expanding label matrix $\hat{L}$ can be expressed as:

(7) $$\hat{L}={\left[\begin{array}{l} \begin{array}{ccc} {\hat{l}}_{1,1} & \cdot \cdot \cdot & {\hat{l}}_{1,v^{\prime }}\\ \cdot \cdot \cdot & \cdot \cdot \cdot & \cdot \cdot \cdot \\ {\hat{l}}_{x,1} & \cdot \cdot \cdot & {\hat{l}}_{x,v^{\prime }} \end{array}\\ \underset{\begin{array}{l} \mathrm{each}\;\mathrm{row}\;\mathrm{is}\;0\;\mathrm{from}\\ \mathrm{row}\;x+1\;\mathrm{to}\;\mathrm{row}\;v^{\prime } \end{array}}{\underset{︸}{\begin{array}{ccc} 0 & \cdot \cdot \cdot & 0\\ \cdot \cdot \cdot & \cdot \cdot \cdot & \cdot \cdot \cdot \\ 0 & \cdot \cdot \cdot & 0 \end{array}}} \end{array}\right]}_{\left(v^{\prime }\cdot v^{\prime }\right)},x<v^{\prime }\leq K-x$$

where ${\hat{l}}_{i,j}$, $1\leq i\leq v^{\prime }$, $1\leq j\leq v^{\prime }$, ${\hat{l}}_{i,j}=0$ $x<i\leq v^{\prime }$.

The adjacency matrix of the paired D2D users can be expressed as:

(8) $$A={\left[\begin{array}{ccc} a_{1,1} & \cdot \cdot \cdot & a_{1,m}\\ \cdot \cdot \cdot & \cdot \cdot \cdot & \cdot \cdot \cdot \\ a_{m,1} & \cdot \cdot \cdot & a_{m,m} \end{array}\right]}_{\left(m\cdot m\right)},m=min\left(x,K-x\right)$$

There is only one 1 in each row and each column, which means that $a_{i,j}=1$ and $a_{h,c}=1$ when i≠h,j≠c. The rest of the elements are all zero. Hence users $U_{i},U_{h}$ of *Y*_1_ pair with $U_{j},U_{c}$ of *N*_1_, respectively.

## 3. Analysis of System Energy Efficiency

Different users have various requirements regarding the same service and one user has different requirements for different services. These differences lead to differences of energy consumption because of the outage probability, which is affected by requirements of QoS in terms of delay and data rate. Hence, it is necessary to take delay and data rate into account when analyzing link outage probability to examine system energy efficiency.

### 3.1. Link Outage Probability

The link is supposed to interrupt when delay $\tau_{\min}$ is bigger than threshold $\tau_{th}$ or data rate *R* is higher than the maximum data rate *R_m_*, which is defined by the Shannon Equation. Hence, the outage probability can be expressed as $P_{out}$ = $1-P\left\{R\leq R_{m},\tau_{\min}\leq \tau_{th}\right\}$, where $R_{m}=B\log_{2}\left(1+Pow{\left|h\right|}^{2}/N_{0}B\right)$, $\tau_{\min}=\tau_{t\min}+\tau_{r\min}+\tau_{p}$. $N_{0}$ is single side band noise power spectral density; *h* is channel gain and ${\left|h\right|}^{2}$ follows independent exponential distribution whose variance is $1/\lambda^{2}$ [17]. *B* is bandwidth and Pow is transmission power. $\tau_{\min}=\tau_{t\min}+\tau_{r\min}+\tau_{p}$. $\tau_{t\min}$, $\tau_{r\min}$ and $\tau_{p}$ are minimum transmission delay, reception delay and propagation delay, respectively. We ignore the propagation delay because of the short distance and suppose that the transmission delay is equal to the reception delay. $\tau_{t\min}=\tau_{r\min}=D/R_{m}$. *D* is the length of the packet. The outage probability $P_{out}$ can be expressed as:

(9) Pout={1−exp(−2RB−1r¯⋅λ),R≥2Dτth1−exp(−22DτthB−1r¯⋅λ),R≤2Dτthr¯=PowN0B

The energy consumption E of sharing a data packet between two D2D users includes transmission energy $E_{t}$, reception energy $E_{r}$ and procession energy $E_{p}$. We suppose that $E_{tr}=E_{t}+E_{r}=2E_{t}=2E_{r}$, so $E=E_{tr}+E_{p}$ because $E_{t}=D\cdot E_{bit}/(1-P_{out})$, and the total energy consumption of each link can be expressed as $E=2D\cdot E_{bit}/P_{pass}+E_{p}$, where $E_{bit}$ is the consumed energy for 1 bit.

### 3.2. System Energy Efficiency

In order to analyze the energy efficiency of the whole cooperation process of sharing data *L*, we have to sum up the energy consumption of each link according to the cooperation scheme analyzed in Section 2. We suppose that cluster head *S* divides data *L* into *n* data packets and share with *K* D2D users within the cluster. The length of each data packet is $D_{d}$ and $L=\sum_{d=1}^{n}D_{d}$. *S* broadcasts data packet $D_{d}$ to $x_{d}$ D2D users first and then these $x_{d}$ D2D users will be the relaying D2D users to relay data packet to the rest of $K-x_{d}$ D2D users. 

There are two types of links within a cluster including head-to-member links and member-to-member links, namely, $S-to-U_{m}$ and $U_{m}-to-U_{j}$. The outage probabilities of $S-to-U_{m}$ and $U_{m}-to-U_{j}$ can be expressed as:

(10) Pm−out={1−exp(−2RBm−1r¯m⋅λ),R≥2Ddτm−th1−exp(−22Ddτm−thBm−1r¯m⋅λ),R≤2Ddτm−thr¯m=PowmN0Bm,d∈(1,⋅⋅⋅,n),m∈(1,⋅⋅⋅,xd)

(11) Pj−outm={1−exp(−2RBjm−1r¯jm⋅μ),R≥2Ddτj−thm1−exp(−22Ddτj−thmBjm−1r¯jm⋅μ),R≤2Ddτj−thmr¯jm=PowjmN0Bjm,d∈(1,⋅⋅⋅,n),m∈(1,⋅⋅⋅,xd),j∈(xd+1,⋅⋅⋅,K)

Hence the energy consumption of *S* sending data packet $D_{d}$ to D2D user $U_{m}$ can be expressed as $E_{m}=2D_{d}\cdot E_{m-bit}/\left(1-P_{m-out}\right)+E_{mp}$, where $E_{mp}$ is the energy consumption of $U_{m}$ for processing data packet $D_{d}$. Thus, the total energy consumption of *S* for broadcasting data $D_{d}$ to $x_{d}$ D2D users can be expressed as:

(12) ES→Ud=∑m=1xdEm=∑m=1xd(2Dd⋅Em−bit1−Pm−out+Emp)

The energy consumption of D2D user $U_{m}$ for sending data packet $D_{d}$ to D2D user $U_{j}$ can be expressed as $E_{j}^{m}=2D_{d}\cdot E_{j-bit}^{m}/\left(1-P_{j-out}^{m}\right)+E_{jp}$, where $E_{jp}$ is the energy consumption of $U_{j}$ for processing data packet $D_{d}$.

The total energy consumption of *K* D2D users for sharing data packet $D_{d}$ should be analyzed in two situations:

**A.** $a=1,0\leq b<x_{d}$. According to the adjacency matrix of the paired D2D users of $G_{1}\left(Y_{1},N_{1}\right)$, the corresponding energy consumption matrix can be expressed as:

(13) $$E={\left[\begin{array}{ccc} E_{1}^{1} & \cdot \cdot \cdot & E_{b}^{1}\\ \cdot \cdot \cdot & \cdot \cdot \cdot & \cdot \cdot \cdot \\ E_{1}^{x_{d}} & \cdot \cdot \cdot & E_{b}^{x_{d}} \end{array}\right]}_{\left(x_{d}\cdot b\right)}$$

where $E_{j}^{m}$ is the energy consumption for relaying data packet from $U_{m}$ to $U_{j}$. $E_{j}^{m}=0$ means that $U_{m}$ does not relay the data packet to $U_{j}$. There is only one member bigger than zero in each column and up to one member who is bigger than zero in each row. Furthermore, there are b rows where there is only one member bigger than zero in each row and the rest of $x_{d}-\left(K-x_{d}\right)$ are all zeros. In other words, if $E_{j}^{m}\neq 0$ and $E_{n}^{i}\neq 0$ only when m≠i and j≠n. The total energy consumption of cooperation between $Y_{1}$ and $N_{1}$ can be expressed as:

(14) EU→Ud1=∑m=1xd∑j=1K−xdEjm

where $E_{j}^{m}=0$ only when $a_{m,j}=0$.

**B.** $a>1,0\leq b<x_{d}$. D2D users will be paired by using Kuhn-Munkres algorithm for *a* + 1 times. The adjacency matrixes of the paired D2D users can be expressed as:

(15) Bg′=[b1,1′(g)⋅⋅⋅b1,xd′(g)⋅⋅⋅⋅⋅⋅⋅⋅⋅bxd,1′(g)⋅⋅⋅bxd,xd′(g)](xd⋅xd),g∈(1,⋅⋅⋅,a)

(16) Ba+1′=[b1,1′(a+1)⋅⋅⋅b1,b(a+1)⋅⋅⋅⋅⋅⋅⋅⋅⋅bb,1′(a+1)⋅⋅⋅bb,b′(a+1)]b⋅b

There is one 1 in each row and each column. In other words, bm,j′(g)=1 and bi,n′(g)=1 only when m≠i,j≠n, g∈(1,⋅⋅⋅,a+1). The $B_{a+1}^{\prime }$ will be an empty matrix if *b* = 0. Thus, the energy consumption matrixes of paired D2D users of $G_{g}\left(Y_{g},N_{g}\right)$ can be expressed as:

(17) Eg=[E11(g)⋅⋅⋅EK−g⋅xd1(g)⋅⋅⋅⋅⋅⋅⋅⋅⋅E1xd(g)⋅⋅⋅EK−g⋅xdxd(g)](xd⋅(K−g⋅xd)),g∈(1,⋅⋅⋅a+1)

There is only one member bigger than zero in each row and each column and there are $x_{d}$ columns, where there is only one member bigger than zero in each column. The rest of the $\left|\left(K-g\cdot x_{d}\right)-x_{d}\right|$ columns are all zeros. In other words, Ejm(g)≠0 and Eni(g)≠0 when m≠i,j≠n. The total energy consumption of cooperation between $Y_{1}$ and $N_{1}$ can be expressed as:

(18) EU→Ud2=∑g=1a+1(∑m=1xd∑j=1K−g⋅xdEjm(g))

Ejm(g) represents the energy consumption of the cooperation between user $U_{m}$ and $U_{j}$. $U_{m}$ and $U_{j}$ are paired by using the *g*th Kuhn-Munkres.

Hence, the total energy consumption of sharing one packet *D_d_* among *K* users can be expressed as:

(19) Ed={ES→Ud+EU→Ud1, xd≥K−xdES→Ud+EU→Ud2, xd<K−xd

If *S* divides data *L* into *n* packets and the length of each packet is *D_d_*, the total energy consumption of sharing *L* with *K* users can be expressed as

$$E_{L}=\sum_{d=1}^{n}E_{d}$$

The energy efficiency will be expressed as:

(20) $$EE=E_{L}^{\prime }/E_{L}$$

where $E_{L}^{\prime }$ represents the total energy consumption when all the outage probabilities of links are 0.

## 4. Simulation Results

In order to achieve D2D multi-user cooperation with a high energy efficiency in different communication environments, the relationships among the number of cooperative users *K*, the length of shared data *L*, the number of date packets *n*, and energy efficiency are studied in this section. We evaluate the system performance in terms of link outage probability and system energy efficiency.

Note that the bandwidth consumed in the D2D communication is 5 MHz and single side band noise power spectral density *N*_0_ is −174 dB/Hz. We suppose that all channel gains followed independent exponential distribution (IED) where variance is 1. The packet error rate can be calculated according to the SNR. We adopt binary quadrature amplitude modulation (QAM) in the Additive Gaussian White Noise (AWGN) channel. Other simulation parameters are listed in Table 1.

**Figure 3:**
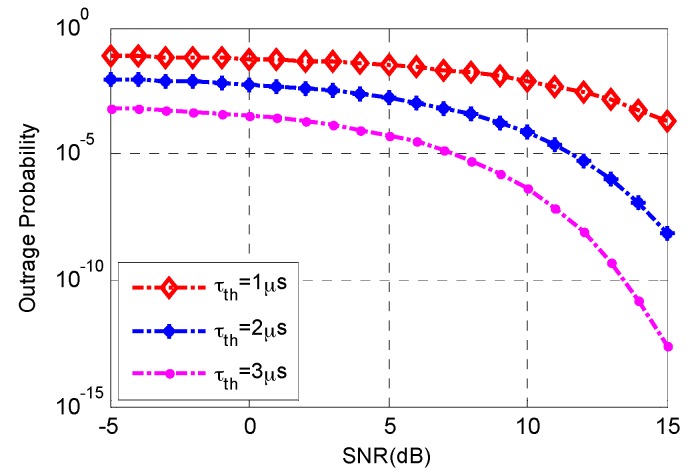
Relationships between the average SNR, average delay threshold τth of D2D link and outage probability, *L* = 512 bits, *K* = 100, *n* = 6.

Figure 3 shows the relationships between the average SNR, average delay threshold of D2D link and outage probability. We suppose that the total length of data is *L* = 512 bits, the number of D2D users within cluster is *K* = 100, and the number of data packets was *n* = 6. Figure 3 shows that the outage probability decreased as average SNR increased since the higher SNR indicates better channel quality, which is conducive to successful transmission. The higher delay constraint led to higher outage probability since the link is interrupted when the delay is beyond the maximum delay the D2D user willing to tolerate. 

**Figure 4:**
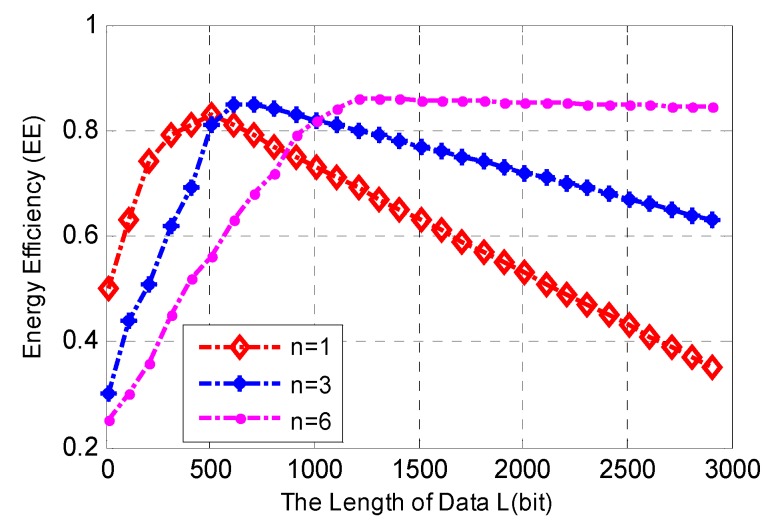
Relationships between the total length of data *L*, the number of data packets *n* and the energy efficiency (EE), and *K* = 100, SNR = 10 dB, τth = 2 μs.

Figure 4 shows the relationships between the total length of data *L*, the number of data packets *n* and the energy efficiency. The number of D2D users within the cluster is *K* = 100, the average SNR of D2D link is 10 dB, and the average delay threshold of D2D link is τ_th_ = 2 μs. Figure 4 shows that when the total length of data *L* is less than a certain value, the energy efficiency of one data packet is higher than the others. This behavior can be attributed to excessive data packets leading to more extraordinary relaying energy consumption on overhead, which wastes energy rather than improving energy efficiency.

Meanwhile, the energy efficiency of a six data packet is higher than that of the others when the total length of data *L* is larger than a certain value. This is due to the shorter data packet, which reduces the outage probability and ensures a reliable transmission for a long data stream.

**Figure 5:**
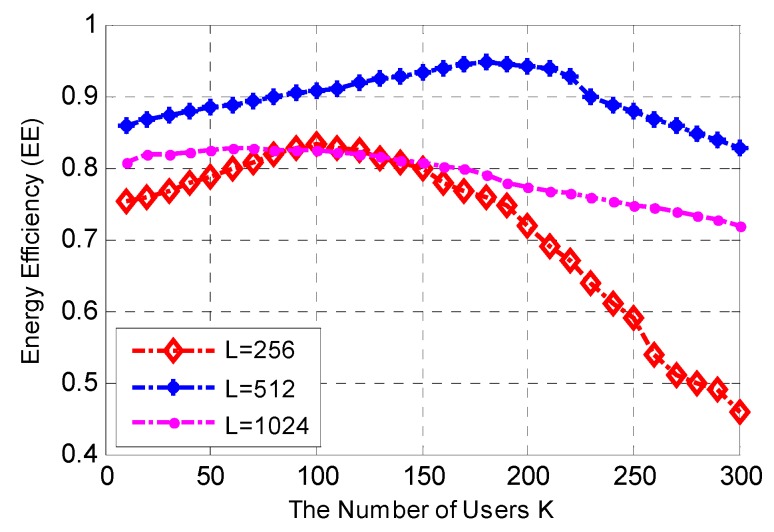
Relationships between the number of cluster D2D users *K*, the total length of data *L* and energy efficiency, *n* = 6, SNR = 10 dB and τth = 2 μs.

Figure 5 shows the relationships between the number of D2D users within cluster *K*, the total length of data *L* and energy efficiency. The number of data packets is *n* = 6, the average SNR is 10 dB and the average delay threshold is τ_th_ = 2 μs. Figure 5 shows that the energy efficiency of the proposed D2D multi-user cooperation scheme for the case of *L* = 512 bits outperforms other cases in the AWGN environment. This occurs when the number of data packets *n* is constant as the excessive long data sequence leads to long data packets creating a high outage probability, which results in decreasing energy efficiency. Meanwhile, when the data sequence is short, excessive data packets leads to wasted energy for relaying overhead.

Furthermore, Figure 5 also shows that for the case of *L* = 1024 and *L* = 512, the energy efficiency almost keeps stable when *K* increases. For the case of *L* = 216, when the number of cluster D2D users *K* is smaller than a certain value, energy efficiency rises very slowly with the increase of *K*, but also decreases quickly when *K* is larger than a certain value. This is because the amount of consumed energy for relaying excessive short data packets with constant overhead to many D2D users is excessive.

From the above analysis, it is obvious that energy efficiency can reach a peak value at certain *K* or *L*. Hence, it is possible to achieve high energy efficiency in different communication environments where multiple D2D users cooperate by coordinating the number of cooperative users *K*, the length of shared data *L*, and the number of date packets *n*.

## 5. Conclusions

D2D communication has great potential in handling problems within complex heterogeneous networks because of its strong flexibility, which has seen wide attention in recent years. D2D multi-user cooperation is considered as an important way of improving spectrum efficiency, throughput and energy efficiency, and is suitable as a relaying approach since D2D users can establish connections flexibly with self-organization and direct communication. Although D2D is widely applied in many networks to help enhance spectrum efficiency, capacity or coverage, etc., the research on cooperation among multiple D2D users is still in its infancy. In this paper, we have proposed a cooperation scheme for multiple D2D users. In order to ensure high QoS without expending too much energy by the D2D users, we consider both energy availability and the SNR of each cooperative user and introduce the Kuhn-Munkres algorithm to help select relaying D2D users. The Kuhn-Munkres algorithm is widely applied in solving MWM problems, and is good at handling matching problems among multiple users. It may be used in the future to solve the spectrum allocation problem, which has been ignored in this paper.

## Figures and Tables

**Table 1:** Simulation Parameters.

Parameters	Values
Distances between D2D users (m)	10–25
Transmission power (dBm)	15–30
Average SNR of D2D link (dB)	10
Total length of data L (bits)	256, 512, 1024
Average delay threshold τth (μs)	1, 2, 3
The number of D2D users within cluster K	10–300
The number of data packets n $\tau_{i\min}^{n}$	1, 3, 6
Transmission power of cluster head S (dBm)	24
Constant overhead of each data packet (bits)	10
